# Sketches in Midwifery Practice

**Published:** 1857-11

**Authors:** Walter Channing


### SELECTIONS.

*Sketches in Midwifery Practice. By Walter Channing, M.D.*

*Sept. 9th.*—I was called in consultation in Mrs. ——'s case, about noon this day. I found her sitting in a chair, if that could be called sitting which was resting the limbs against a chair seat, with the least possible bending of the body upon them. She said that she could not lie down for a moment, so embarrassing to breathing was that position. The difficulty arose from the anasarcous enlargement, hardness and stiffness of the limbs. It was not possible to make indentation by pressure any way. At the wrinkles about the joints, large ridges or rings surrounded them, and betweon muscles and faecia, elsewhere, the water filled and projected the integuments in distinct tumors. One on the upper and inner part of the left thigh promised to embarrass any operation which might be in hand. Her age is 19, and this is her first labor. It began six days before my visit; continued two days, making fair progress, and then ceased, leaving the head at rest in the bony outlet. Her size was noticeable. I have seen many large persons under her circumstances, but she excelled them all. Since the moment the pains ceased, no uterine action has manifested itself, and for four days her life has been a continuous misery. The pulse was rapid, skin hot, she was sleepless, and the functions of the bladder and rectum were embarrassed. Attempts have been made to produçe uterine action, but in vain.

In consultation, the sole question regarded artificial delivery, and upon due consideration it was decided that an attempt should be made. After much difficulty, and suffering on her part, Mrs. —— was laid on her bed, and her case being stated to her, she agreed to do her best to remain upon it. The forceps were first tried, and, it was thought, faithfully, but with no effect. The head was reduced, and after long-continued effort the child was removed. The abdomen was examined, and it seemed just as large, just as full as it did before the delivery was effected. It was clear that another child, at least, remained in the womb. It was agreed that this should be delivered by turning. This was done, during full etherization (this state was sustained from the first attempt at delivery), and after a prolonged and decided effort delivery was accomplished.

Now, during these operations the womb remained perfectly quiet. I could not discover the least mark of contraction. When the first child was born, the part of the womb it had occupied remained perfectly soft and empty, while the tumor formed by the second child was at the highest point in the abdomen. After the second birth, the womb preserved its remarkable quietude. The placentas remained adherent, not a single drop of blood was shed. After waiting for action to occur, and having waited in vain, the placentas were taken away. While this was accomplishing, and the womb necessarily much irritated by the manipulations, the organ remained perfectly at rest. The cavity which had been filled by two fœtuses, weighing together twenty-two pounds, one of them having been subjected to craniotomy, which certainly had not increased its weight—this cavity remained just as large as before delivery, and was absolutely embarrassing by its extent. The placentas were slowly raised, for the flaccid womb yielded too readily to pressure to allow any more than a peeling process to be practised. No hæmorrhage accompanied or followed the operation. A napkin was useless.

For two days after delivery there were involuntary rectal discharges, and the catheter was used for the same length of time. After these accidents, convalescence proceeded slowly but without interruption, and recovery was perfect.

*Sept. 13th.*—This was Mrs. ——'s fifth labor. The four preceding were natural. I was called to see her between 11 and 12 p.m., and learned she had been ill for many hours. Uterine contractions had been strong, and had brought the head to the inferior strait. About four hours before my visit, her first physician—for two were in attendance when I was called—had discovered a tumor of great size projecting from the abdomen with the umbilicus for its central point, and tympanitic to a degree which to him had never been equalled in puerperal fever or in any other disease. At first it was thought it might depend upon some condition of the bladder, but the catheter shed no light upon it. There was no precedent tenderness of abdomen, and no constitutional condition which comes of inflammation anywhere.

I found this state of things as described by the physician who had come for me, or rather to borrow a long catheter, and who asked me to return with him. The tumor, for so it had been called, was found as tense as the integuments allowed it to be. These were made so thin by distension that they seemed reduced to a mere membrane, suggesting the idea that the skin would give way under the least outward pressure upon it. But that it would be trenching upon another department of literature, an illustration in the way of comparison might, by a figure of rhetoric, make this extraordinary abdominal affix much more readily understood than can a merely verbal description. There were no uterine contractions. These had ceased for some time. Mrs. —— was exhausted and entirely hopeless.

Upon examination, the head was found as described, closely impacted in the pelvis. The anterior lip of the womb was hanging down greatly enlarged, reaching almost to the external organs. It was firm, hard, compressed between the fœtal head and symphsis, its free part being moveable, thick, and having a rounded edge. At first it was thought it might be pressed up between the head and pelvis, and thus very much facilitate delivery. It did actually seem to recede by pressure, but this turned out to be nothing more than a mechanical shortening of it, for as soon as pressure ceased it came down into its former place. It was clear that delivery could only be effected by mechanical means, and it was quite as clear that the abdomen was rapidly growing larger. The long catheter brought away no water.

The forceps were tried—at first Davis'—but their breadth and large curvature made it impossible to introduce them without a degree of pressure upon the enlarged anterior lip of the womb, which was thought anything but safe. Davis' instrument was tried, because one of the physicians present had brought it with him, and it was lying on the table. Prof. Hodge's, of Philadelphia, were now used, and were introduced with ease and success. Except when the cranium is very low, I always use Prof. H.'s. At other times Hamilton's are preferred in the other situations of the head. I brought one of Prof. Hamilton's instruments from Edinburgh more than forty years ago, and have never known it fail in proper cases.

As soon as the first branch was introduced, a great blast of most fœtid air rushed from the vagina, and also a quantity of water of the same odor. The adventitious abdominal tumor at once disappeared, and the hand now rested upon the uterine walls, through the thin and relaxed abdominal walls. No contractions occurred upon the introduction of the forceps, or accompanied its use. Delivery was accomplished with great difficulty, the getting away of the trunk requiring much more effort than did the head. The only way, in fact, of accomplishing this task, was by carrying the blunt hook into the axillæ, first one and then the other. The placenta was adherent, and was removed by the hand.

Moderate reaction followed. On the third day some castor oil was given, which operated kindly. The milk came. On the sixth day the pulse was found quickened, and much weakness was complained of. There were no symptoms of peritonitis, nor of other local inflammatory trouble. Sinking came on, and rapidly increased, and on the tenth day from delivery Mrs. —— died. Her medical attendant, who communicated these facts to me, could assign no internal cause for their occurrence. The room in which Mrs. —— was confined was very small, without ventilation. Its door opened into another small room, in which was a cooking stove, and in which the family of four children, etc., lived. The weather was unusually hot for the season. These circumstances were certainly very unfavorable, and one would think quite sufficient to disturb the convalescence which had so kindly advanced, and promised so happy a result.

A question of the cause of the formation of gas in the womb may arise. I have met with no such case before, and I cannot call to mind its like from the books. It shows how closely was the head in contact with the vagina and pelvis, for the smallest opening would have allowed of the escape of the gas. Air might have entered the womb when the membranes broke, and a portion of the liquid amnii had come away. So it may do in other cases. But who has met with such an instance; and without the presence of atmospheric air, how would the chemical changes referred to have been produced? The dead fœtus may become emphysematous. This was not the case in Mrs. ——'s child; and if it had been, how could the air escape through the unbroken integuments? The tumor occurred suddenly, and rapidly increased in size. From its feel it seemed utterly impossible that the womb should have been so thinned as to have aided in forming the walls that contained the gas. Why were not the relations between the afterbirth and the womb changed by such an extreme expansion of its substance? The placenta does not grow *less* under such contractions as separate it; if so, it would have one of the properties of the cotyledons, which nourish the young ruminant during its intrauterine life. The placenta is separated, because the womb to which it is attached grows *smaller*, while itself preserves its natural size. The *less* cannot contain the *greater*, unless morbid adhesion exists.

However the reasoning may be, the fact remains. The womb was distended with gas, to the apparent threatening of its disruption. This gas was decomposed, or rather that from which it was produced was decomposed, and set it free. This gas was certainly contained in the womb. The marked convalescence continuing uninterrupted for a week, with the regular performance of all the functions, proves, I think, that no such lesion existed as would produce either disorder, disease or death. It is seriously regretted that an examination after death was not made. The position of the patient—prejudices, religious and others, doubtless prevented this. I did not see Mrs. —— after her delivery.

*Sept. 29th.*—This was Mrs. ——'s fourth labor. I had attended her in all of them, with her family physician. Her first labor was natural. The second was instrumental. The third, natural; and the fourth, or last, was instrumental.

I was called to see Mrs. —— at the date above, between 7 and 8 a.m. Uterine contractions strong. Began at 6 a.m., two hours before my arrival. Examination showed the first stage of labor perfectly accomplished. Os uteri had disappeared. Head fairly in the inferior strait, and advancing during contractions. In about an hour progress ceased. Great suffering, strong bearing down, but conscious of no progress. This state continued about four hours, and precisely imitated the state of things in the second labor, when the forceps were used, and in a few minutes most favorably completed delivery. A single effort now with the instrument brought the head beyond the obstacle to its progress, and its continued advance made other than natural effort unnecessary. As the head emerged, the instrument was removed. Mother and child recovered without accident.—*Boston Med. and Sur. Journal.*

